# Case Report: Probable blinatumomab-associated lung injury in a pediatric patient with B-ALL

**DOI:** 10.3389/fphar.2026.1973818

**Published:** 2026-10-21

**Authors:** Xingxing Feng, Tiantian Yin, Juan Han, Fen Zhou

**Affiliations:** Department of Pediatrics, Union Hospital, Tongji Medical College, Huazhong University of Science and Technology, Wuhan, China

**Keywords:** B Cell acute lymphoblastic leukemia, blinatumomab, lung injury, pediatric, rechallenge

## Abstract

Blinatumomab is a bispecific T Cell engager targeting CD3 and CD19. Initially approved for relapsed/refractory B Cell acute lymphoblastic leukemia (B-ALL), it was later extended to first-line B-ALL therapy. Despite its high efficacy, blinatumomab carries risks of adverse effects, including cytokine release syndrome and neurotoxicity. Blinatumomab-associated lung injury is exceptionally rare. We report a pediatric B-ALL patient who developed drug-induced lung injury following induction chemotherapy, with recurrence of lung injury after blinatumomab administration. The patient presented with dyspnea, hypoxemia, and radiographic findings suggestive of interstitial lung injury. After excluding infectious and other non-infectious etiologies, we considered blinatumomab-associated lung injury as the most likely diagnosis. Symptoms improved markedly following corticosteroid therapy. Clinicians should be alert to blinatumomab-associated lung injury, achieve early recognition and administer glucocorticoid intervention. A dose-escalation strategy facilitates successful rechallenge.

## 1 Introduction

B Cell acute lymphoblastic leukemia (B-ALL) is the most common childhood malignancy, representing approximately 30% of pediatric cancers (Hunger et al., 2012). Over the past decades, multi-agent chemotherapy and refined risk stratification have improved cure rates for pediatric B-ALL to over 90% (Hunger et al., 2012). While conventional chemotherapy remains fundamental, immunotherapy has now reshaped the treatment landscape. Initially approved for relapsed/refractory B-ALL, blinatumomab has now been incorporated into first-line regimens (Gupta et al., 2025; Sidaway, 2025).

Blinatumomab can activate T-cell-mediated cytotoxicity against CD19-positive tumor cells by concurrently binding CD19 on B Cells and CD3 on T Cells (Nagorsen et al., 2012). Blinatumomab shows strong antineoplastic activity in B-ALL but can cause adverse effects. The most common are cytokine release syndrome (CRS) and neurotoxicity, while rare events such as pneumatosis intestinalis have been reported (Gao et al., 2025; Kim et al., 2021). With expanding clinical use, heightened vigilance regarding potential adverse events is warranted. Critically, rare cases of blinatumomab-associated lung injury have been described in the literature. We report a pediatric patient with B-ALL who developed lung injury after induction chemotherapy, which recurred during blinatumomab therapy. This article focuses on the differential diagnosis between CRS and drug-induced interstitial lung disease (DIILD) in lung injury associated with blinatumomab, and describes the clinical experience of successful blinatumomab rechallenge using a graded dose-escalation strategy.

## 2 Case presentation

We report a 10-year-old male patient with low-risk B-ALL with *EP300::ZNF384* fusion. The patient was treated according to the Chinese Children’s Cancer Group Acute Lymphoblastic Leukemia 2025 protocol and successfully completed induction therapy. The patient achieved complete remission and absence of the *EP300::ZNF384* fusion transcript. After induction therapy, treatment with blinatumomab was scheduled for this pediatric case.

On day 11 after completion of the CAT regimen during induction therapy (cyclophosphamide, cytarabine, mercaptopurine), the patient developed progressively worsening dyspnea, hypoxemia, and moist rales, without fever or hypotension, representing the first episode of lung injury. Although the patient’s neutrophil count was normal and both infection markers and microbiological tests were negative, we initially considered infection as the primary cause and initiated empirical anti-infective therapy. Further investigations revealed normal cardiac function and chest computed tomography (CT) findings, fiberoptic bronchoscopy showed no abnormalities, and metagenomic next-generation sequencing (mNGS) of bronchoalveolar lavage fluid detected no pathogens (Figure 1). However, the patient’s symptoms did not improve with antibiotics. Accordingly, we excluded infection, cardiogenic factors, and structural lung abnormalities, and recent bone marrow evaluation and chest CT also ruled out pulmonary tumor infiltration. Given the temporal relationship with the recent CAT regimen chemotherapy, we considered DIILD (Figure 2). After antibiotic discontinuation, the patient received high-flow nasal cannula oxygen, budesonide nebulization, and intravenous methylprednisolone, with gradual clinical improvement. No recurrence was observed during the stepwise reduction of oxygen support and tapering of corticosteroids. A follow-up chest CT scan was subsequently performed, and the results remained negative.

**FIGURE 1:**
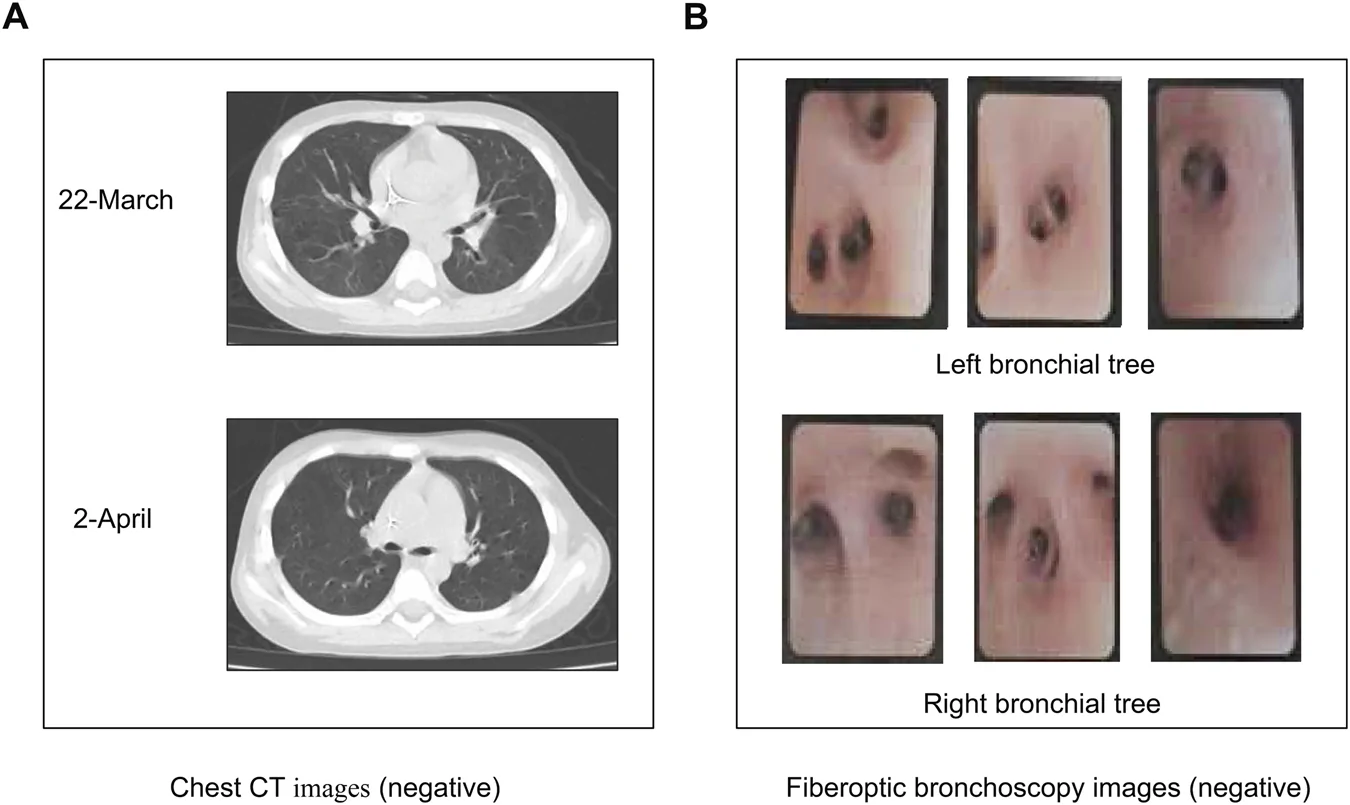
Imaging findings during the first episode of lung injury. **(A)** Chest CT images; **(B)** Fiberoptic bronchoscopy images.

**FIGURE 2:**
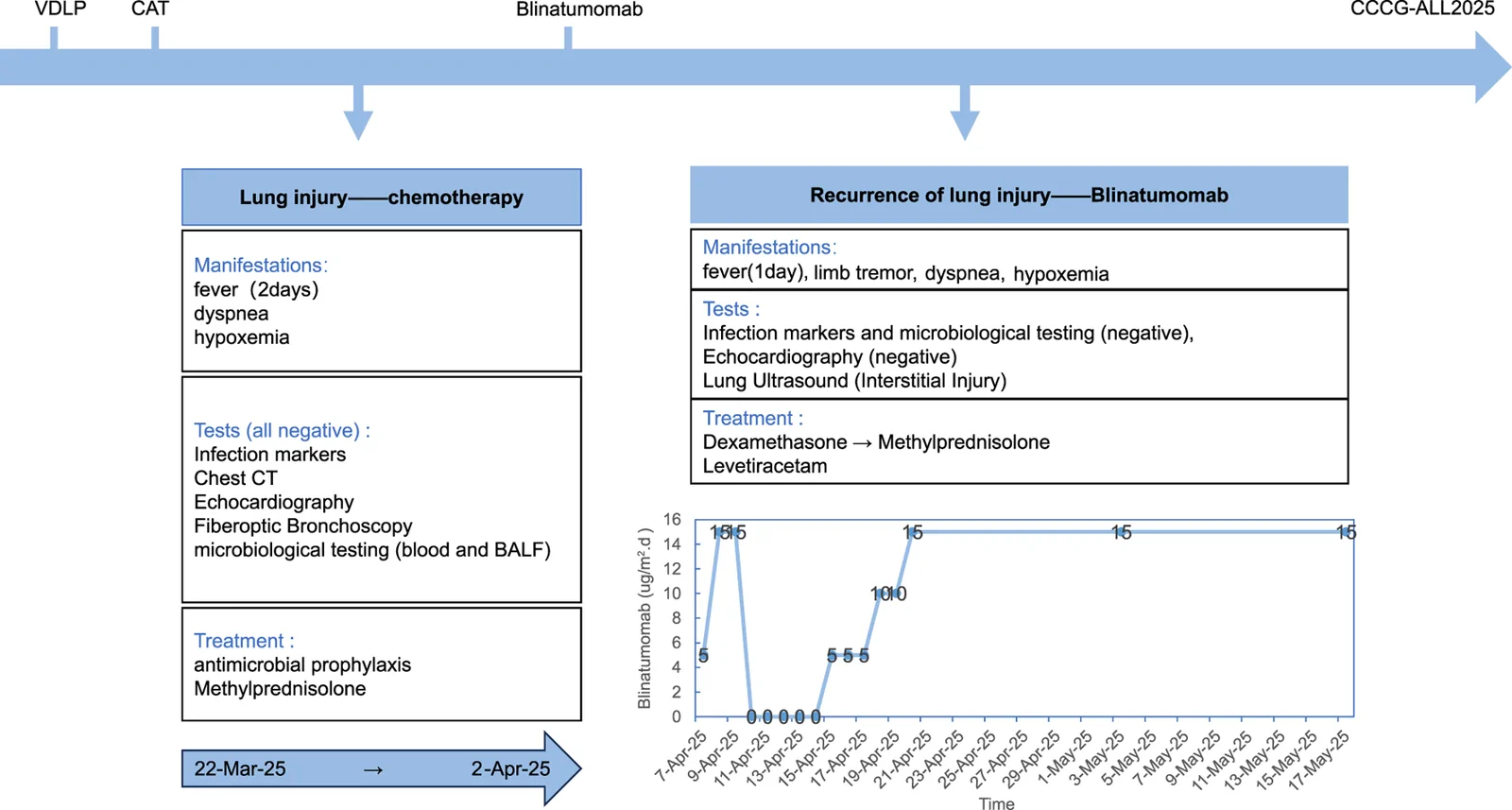
Detailed information on the treatment process of chemotherapy and blinatumomab. VDLP: vincristine (VCR), daunorubicin (DNR), L-asparaginase, and prednisone; CAT: cyclophosphamide (CTX), cytarabine (Ara-C), and mercaptopurine (6 MP); CCCG-ALL2025: Chinese Children’s Cancer Group - Acute Lymphoblastic Leukemia 2025 Protocol; BALF: Bronchoalveolar Lavage Fluid.

The second episode of lung injury occurred during blinatumomab treatment, which was initiated on April 7 (Figure 2). The patient tolerated both the initial 5 μg/m^2^ dose and the escalation to 15 μg/m^2^ on day 2 without complications. However, during continued 15 μg/m^2^ dosing on day 3, the patient developed low-grade fever (temperature: 37.5 °C) accompanied by perioral and bilateral hand myoclonus, markedly exacerbated by voluntary movement. Comprehensive evaluation (microbiological tests, inflammatory markers, electrolytes, and cytokine profiling) revealed no abnormalities, thereby excluding potential infectious etiologies and electrolyte imbalances (Supplementary Table S1). These manifestations were initially attributed to common blinatumomab-related adverse effects (pyrexia and tremor), although CRS could not be excluded. The patient was treated with dexamethasone (0.3 mg/kg) and levetiracetam (20 mg/kg), while blinatumomab was maintained at the dose of 15 μg/m^2^. On day 4 of blinatumomab therapy (approximately 48 h after full-dose blinatumomab, 15 μg/m^2^), the patient developed tachypnea (Respiratory Rate, RR: 36 breaths/min) and tachycardia (Heart Rate, HR: 134 beats/min), with coarse crackles on pulmonary auscultation. The condition progressed to dyspnea and hypoxemia (Peripheral Capillary Oxygen Saturation Percentage, SpO_2_ < 95% on ambient air), resembling previous drug-induced lung injury. Laboratory tests, including C-reactive protein, procalcitonin, and serial blood cultures, further excluded infectious causes. Cardiac enzyme levels and echocardiography were both unremarkable, ruling out cardiac etiologies. Lung ultrasound revealed diffuse bilateral B-lines, suggesting interstitial lung injury (Table 1). Pulmonary function testing was not performed due to poor patient cooperation; additionally, chest CT was not repeated given radiation exposure concerns. Given the current treatment, similar clinical presentation, evidence of interstitial lung injury, and a Naranjo score of 6 (Supplementary Table S2), blinatumomab-associated lung injury was suspected, although CRS could not be completely excluded.

**TABLE 1:** Results of lung ultrasound examinations during blinatumomab therapy.

Sites	Location	14-Apr-2025		19-May-2025	
​	​	B line	Consolidation	B line	Consolidation
Left lung	L1	Few	Not seen	Not seen	Not seen
	L2	Few	Not seen	Few	Not seen
	L3	Few	Not seen	Few	Not seen
	L4	Few	Not seen	Not seen	Not seen
	L5	Few	Not seen	Not seen	Not seen
	L6	Few	Not seen	Not seen	Not seen
Right lung	R1	Not seen	Not seen	Few	Not seen
	R2	Few	Not seen	Few	Not seen
	R3	Few	Not seen	Not seen	Not seen
	R4	Few	Not seen	Few	Not seen
	R5	Few	Not seen	Few	Not seen
	R6	Few	Not seen	Few	Not seen

Lung ultrasound was performed in six zones per side (L1-L6/R1-R6): 1 = upper anterior, 2 = lower anterior, 3 = upper lateral, 4 = lower lateral, 5 = upper posterior, 6 = lower posterior.

Subsequently, blinatumomab was immediately discontinued, and intravenous methylprednisolone was initiated at 4 mg/kg/day (2 mg/kg every 12 h), combined with nebulized budesonide and supplemental oxygen support; no antibiotics were used during this process. Within 72 h, the patient showed significant clinical improvement, and the corticosteroid dose was tapered to 3 mg/kg/day. After 2 additional days of treatment, symptoms completely resolved and oxygen saturation returned to normal. The corticosteroid was then reduced to 2 mg/kg/day and maintained long-term. During maintenance at 2 mg/kg/day, after discussion with the family, a modified blinatumomab rechallenge was initiated using a graded dose-escalation strategy: starting at 5 μg/m^2^/day (days 1–3), increasing to 10 μg/m^2^/day (days 4–5), and subsequently reaching the target dose of 15 μg/m^2^/day. The patient successfully completed the full 28-day course without recurrence of adverse events (Figure 2). Follow-up lung ultrasound showed significant resolution of B-lines, confirming marked pulmonary improvement (Table 1).

## 3 Discussion

In cancer treatment, interstitial lung injury induced by cytotoxic chemotherapy, targeted therapy, and immunotherapy is classified as DIILD (Skeoch et al., 2018). DIILD has been reported in association with CAR-T cell therapy and antibody-drug conjugates, whereas blinatumomab-associated pulmonary toxicity has rarely been documented to date (Shi et al., 2024; Haas et al., 2023). We report a pediatric patient with B-ALL whose blinatumomab-associated lung injury was most consistent with DIILD, although CRS with predominant pulmonary manifestations could not be ruled out. The lung injury resolved with corticosteroid therapy, and blinatumomab was safely reintroduced using a graded dose-escalation strategy.

The diagnosis of DIILD requires establishing a clear temporal link between the administration of the causative drug and respiratory manifestations, alongside rigorous exclusion of alternative etiologies such as infection (Limper, 2004). In the present case, the first episode of lung injury occurred after CAT regimen chemotherapy. Repeated microbiological tests, imaging studies, and lack of response to anti-infective therapy excluded infectious and alternative etiologies. Combined with the therapeutic response to corticosteroids, this episode was consistent with drug-induced lung injury from chemotherapeutic agents. The second episode of lung injury exhibited a strong temporal and clinical association with blinatumomab: (1) symptoms of lung injury occurred approximately 48 h after full-dose blinatumomab administration; (2) a comprehensive diagnostic workup excluded potential etiologies, including infection, cardiac disease and tumor infiltration; (3) lung ultrasound revealed diffuse bilateral B-lines, consistent with an interstitial injury pattern; (4) the Naranjo adverse drug reaction score was 6 (probable); (5) prompt symptom resolution after blinatumomab discontinuation and initiation of corticosteroids and supportive care (without antibiotics). These findings collectively support a probable causal association between blinatumomab and lung injury. However, because CRS with predominant pulmonary manifestations can overlap clinically with DIILD and is common during blinatumomab therapy, careful differential diagnosis is needed in this case.

Similar cases of pulmonary symptoms occurring shortly after blinatumomab administration have been reported in both adults and children, with some attributed to CRS and others to interstitial lung injury. Shannon described a 47-year-old woman who developed acute severe dyspnea and hypoxemia 7 h after the first blinatumomab infusion, progressing within 24 h to respiratory failure requiring mechanical ventilation and vasopressor support, and ultimately dying from refractory hypoxemia and hypotension (Shannon, 2019). The report lacks detailed documentation of typical CRS features (fever, hypotension, and cytokine elevation), but its fulminant course with systemic involvement was most compatible with severe CRS. Hizal et al. reported a 4.5-year-old boy who developed dyspnea and hypoxemia several days after blinatumomab treatment; chest CT demonstrated extensive interlobular septal thickening, patchy consolidation, and bilateral ground-glass opacities, and the patient improved markedly with corticosteroids; drug-induced interstitial pneumonia was diagnosed (Hizal et al., 2024). In that report, the absence of fever together with unequivocal CT evidence of interstitial pneumonia rendered the attribution to DIILD more convincing.

Pulmonary toxicity induced by blinatumomab includes CRS-related pulmonary manifestations and DIILD, which are clinically challenging to differentiate. Typically, CRS presents earlier with acute systemic inflammation, high fever, elevated inflammatory cytokines, and pulmonary edema on imaging, whereas DIILD occurs relatively later, manifests predominantly as isolated pulmonary involvement, and shows interstitial pneumonia radiologically (Skeoch et al., 2018; Lee et al., 2019). The patient in our report had no systemic symptoms or increased cytokine levels. Lung ultrasound B-lines suggested interstitial lung injury, which also favored DIILD.

Both CRS and DIILD share a common pathogenesis of T Cell activation and inflammatory cascade activation (Shannon, 2019; Zhang et al., 2021). Due to overlapping features and limited value of single time-point temperature or cytokine detection, definitive distinction is often difficult, and such cases can be categorized as immune-related pulmonary toxicity (Lee et al., 2019; Klinger et al., 2012). Initial management consists of immediate interruption of blinatumomab and glucocorticoid administration. Additional interleukin-6 (IL-6) blockade with tocilizumab is indicated for systemic CRS, while DIILD is mainly treated with corticosteroids and respiratory support (Fontes et al., 2024; Choudhry et al., 2018). Decisions regarding permanent discontinuation or treatment rechallenge should be individualized based on toxicity severity, steroid response, imaging changes, and oncological benefits (Skeoch et al., 2018; Lee et al., 2019).

The most clinically instructive aspect of this case lies in the successful rechallenge strategy with blinatumomab. After methylprednisolone treatment, the patient’s symptoms improved and oxygen saturation returned to normal. Although a few B-lines persisted on lung ultrasound, there were no severe abnormalities on pulmonary imaging; blinatumomab rechallenge was therefore initiated under low-dose methylprednisolone maintenance. We used a modified graded dose-escalation regimen, restarting at 5 μg/m^2^/day (days 1–3), increasing to 10 μg/m^2^/day (days 4–5), and finally reaching 15 μg/m^2^/day. The patient successfully completed the full 28-day rechallenge course without recurrence of lung injury. Follow-up ultrasound performed 2 days after completion of the rechallenge course showed further reduction of B-lines to 7 of 12 zones. This may be related to the delayed radiologic recovery and continued stimulation by blinatumomab. The persistence of residual B-lines suggests that even though corticosteroid maintenance prevented recurrence of immune lung injury, subclinical immune stimulation in the lungs may still persist. Blinatumomab currently holds a frontline position in B-ALL therapy, and permanent discontinuation may carry a substantial risk of relapse. After grade 3 (CTCAE v5.0) pulmonary toxicity during blinatumomab therapy, timely recognition and corticosteroid treatment are essential, but the decision to permanently discontinue blinatumomab should be made with caution (Health. NIo and Institute. NC, 2017). In this case, under low-dose corticosteroid maintenance and close monitoring, gradual dose-escalation allowed safe reintroduction of blinatumomab, providing a reference for clinicians facing similar situations (Conte et al., 2022).

In this case, it is also worth noting that the interval between the two episodes of lung injury was relatively short. We hypothesize that the initial chemotherapy-induced lung injury may have rendered pulmonary tissue susceptible to subsequent immune stimulation, thereby heightening vulnerability to blinatumomab-mediated immune injury. This hypothesis is consistent with previous reports identifying pre-existing pulmonary disease as a significant risk factor for DIILD (Skeoch et al., 2018). These mechanisms are currently well established in the context of immunotherapy-related pulmonary toxicity (Zhang et al., 2021).

This study has several limitations. First, due to the child’s poor cooperation and ethical concerns regarding repeated CT radiation exposure, we did not repeat high-resolution CT during the acute phase of lung injury, but instead used lung ultrasound as the primary imaging follow-up tool. Second, given the procedural risks of lung biopsy, pathological specimens were not obtained. In addition, the lack of dynamic cytokine monitoring limited our ability to precisely capture the transient peak. These limitations preclude definitive attribution of the lung injury to CRS versus DIILD, and future similar cases should incorporate these assessments.

In summary, as blinatumomab is increasingly incorporated into frontline therapy for B-ALL, clinicians should remain vigilant for a probable association between blinatumomab and pulmonary toxicity. Due to the overlapping clinical and immunological features of CRS and DIILD, both diagnoses should be considered in patients with pulmonary toxicity associated to blinatumomab. Whether attributed to CRS or DIILD, timely recognition, discontinuation of the suspected agent, adequate corticosteroid intervention, and an individualized graded rechallenge strategy following comprehensive evaluation may help balance antitumor efficacy and treatment safety.

## Data Availability

The original contributions presented in the study are included in the article/Supplementary Material, further inquiries can be directed to the corresponding authors.
