# Supplementary material for: Case Report: Probable blinatumomab-associated lung injury in a pediatric patient with B-ALL
Source: Front Pharmacol. 2026 Oct 21;17:1973818. doi: 10.3389/fphar.2026.1973818 (PMC13731319; doi:10.3389/fphar.2026.1973818)
Supplement: Supplementary file 1 [file Table1.docx]

# Supplementary Material

Table S1 Cytokine levels before and after blinatumomab therapy (two time points)

| Cytokine（pg/ml） | 7-Apr-2025 (Day 1)  Pre-blinatumomab | 10-Apr-2025 (Day 4)  Post-blinatumomab |
| --- | --- | --- |
| IL-2 | 2.34 | 1.50 |
| IL-10 | 6.24 | 1.74 |
| IFN-γ | 1.96 | 1.36 |
| IL-17A | 2.99 | 1.66 |
| IL-6 | 5.68 | 5.14 |
| TNF-α | 2.77 | 1.84 |
| IL-4 | 1.27 | 1.23 |

IL-2: Interleukin-2; IL-10: Interleukin-10; IFN-γ: Interferon-gamma; IL-17A: Interleukin-17A; IL-6: Interleukin-6; IL-4: Interleukin-4; TNF-α: Tumor Necrosis Factor-alpha.

Table S2 Naranjo Adverse Drug Reaction Probability Scale

| Question | Yes | No | Do not know | Selected Answer (√) |
| --- | --- | --- | --- | --- |
| 1. Are there previous conclusive reports on this reaction? | 1 | 0 | 0 | No (0√) |
| 2. Did the adverse event appear after the suspected drug was administered? | 2 | −1 | 0 | Yes (+2√) |
| 3. Did the adverse reaction improve when the drug was discontinued or a specific antagonist was administered? | 1 | 0 | 0 | Yes (+1√) |
| 4. Did the adverse reaction reappear when the drug was readministered? | 2 | −1 | 0 | No (-1√) |
| 5. Are there alternative causes (other than the drug) that could on their own have caused the reaction? | −1 | 2 | 0 | No (2√) |
| 6. Did the reaction reappear when a placebo was given? | −1 | 1 | 0 | Do not know (0√) |
| 7. Was the drug detected in the blood (or other fluids) in concentrations known to be toxic? | 1 | 0 | 0 | Do not know (0√) |
| 8. Was the reaction more severe when the dose was increased, or less severe when the dose was decreased? | 1 | 0 | 0 | Yes (1√) |
| 9. Did the patient have a similar reaction to the same or similar drugs in any previous exposure? | 1 | 0 | 0 | Yes (1√) |
| 10. Was the adverse event confirmed by any objective evidence? | 1 | 0 | 0 | No (0√) |

Total Score Calculation: Definite ADR: ≥9; Probable ADR 5-8; Possible ADR 1-4; Doubtful ADR: ≤0. The total score for the pediatric patient was 6 points.

Table S3 Coagulation factor levels in blinatumomab therapy (24-Apr-2025)

| Coagulation Factor | Levels | Reference Range |
| --- | --- | --- |
| FVIII:C% | 113.8 | 70-150 |
| FIX:C% | 112.9 | 60-150 |
| FXI:C% | 118.2 | 70-120 |
| FXII:C% | 102 | 60-150 |
| FII:C% | 136.8 | 70-120 |
| FV:C% | 158.7 | 70-120 |
| FVII:C% | 98.2 | 55-170 |
| FX:C% | 99.9 | 70-120 |

Figure S1 Changes in coagulation parameters during blinatumomab therapy. A: Prothrombin Time (PT); B: D-dimer; C: Fibrinogen (FIB); D: Activated Partial Thromboplastin Time (APTT).
